# News and Notes

**Published:** 1900-04

**Authors:** 


### *NEWS AND NOTES.*

THE *Providence Medical Journal* is a new periodical from Providence, R. I. The first number contains much excellent matter.

READERS are requested to see the special announcement of Chicago Policlinic and Hospital on page ——, as it contains interesting news.

WE have just received of Fellows Medical Manufacturing Co., 48 Vesey St., New York, a most artistic brochure on the therapeutics of Fellows' Hypophosphites. Write them for a copy.

THE *Post-Graduate*, a monthly medical journal published by the Post-Graduate Medical School in New York, is one of the best journals among our exchanges. It always contains good matter.

THE annual meeting of the Southern Section of the American Laryngological, Rhinological and Otological Society will be held at the Galt House, First and Main, Louisville, Ky., Friday, March 30th, 1900.

THE daily papers announce the extraction of a snake from the throat of a woman near Augusta, Ga. The reptile had been hiding in this mucous nest for two years, but was at last teased from its lair by a Georgia physician.

DR. GEORGE F. BUTLER of this city has been appointed Superintendent of the Sanitarium at Alma, Michigan, to succeed Dr. E. S. Pettyjohn, the appointment to take effect May 1st. Dr. Butler will retain his college connection with the Chicago College of Physicians and Surgeons, and will continue to edit the *Doctors' Magazine.*

THE new Mount Sinai Hospital, now in process of erection, will probably be the most complete in all its details of any in the world. It will occupy the entire block between Fifth and Madison avenues and 100th and 101st streets, and when fully equipped will cost $1,335,000. There will be ten sets of fire-proof buildings, connected by corridors, four stories high, of brick and white marble, accommodating 450 patients.

---

IT is proposed to limit the output of doctors in Russia. The Minister of Education has recently fixed the number of first-year students that will be allowed to enter the various universities. The sum total amounts to 1,095, which does not include the St Petersburg Military Academy, which will take in about 250 students. In striking contrast to the limitation of students in Russia is the effort being made to attract medical students to the University of Buda Pesth. At that place the fees for lectures and classes have been remitted to a considerable number of students, and to those who pay any fees the amount has been much lessened.

---

AT the annual meeting, October 2, of the Detroit Medical and Sanitary Association, the president, Dr. Charles Douglas, in his address, "Teaching the Principles of Nutrition in the Public Schools," said that proper nutrition is the foundation of human development and success. "As the horseman knows that the blue-grass of Kentucky grows the best bone, nerve and muscle in his young animals; as the farmer knows that corn is the best fattening agent for his stock; as the milkman knows that certain kinds of food will give copious returns in milk, and certain other foods produce fat and muscle only; as the florist knows that clay soil grows the best roses, and leaves make the best manure, so must we teach our young the value of individual food in supporting particular parts of the body."—*The Medical Times.*

AN Australian champion cyclist fell forward in his saddle twenty-five yards before the end of a carnival race, and with his feet still moving with the pedals, reached the winning post, when it was discovered he was dead. The story, if authentic, as it appears to be, records another striking instance of the indomitable pluck of athletic men. It shows, moreover, the unerring balance and precision needed in a race, and the instinct that caused the rider, even in the act of dying, to throw himself into and maintain a proper poise. This is the only instance, probably, ever recorded of a race being won by a dead man, and it is said the doctors stated he died during the last lap. Medical cyclists will naturally look forward with interest to learning further details of this most tragic affair.

---

FIFTY tons of candy have been sent to the soldiers in the Philippine Islands by the commissary department of the army during the last three months, and large amounts to the soldiers in Cuba and Porto Rico. This is done upon the advice of the medical as well as line officers of the army, because it is a physiological fact that in the tropics a moderate consumption of confectionery promotes health and satisfies a natural and not unheathful craving of the stomach. Candy was never furnished to the United States army before, although it is commonly used as a ration by the British and French troops in the tropics. The larger part of the shipment are chocolate creams and lemon and other acidulated drops, which are hermetically sealed in one-pound tins of oval shape to fit the pocket of a soldier's uniform. The candy is manufactured in New York especially for the commissary department, and is little more than sugar and lemon or lime juice. It is probable that sugar henceforth will form a part of the soldier's regular ration.

---

DR. KEELEY, the inventor of the "Keeley Cure," died at Los Angeles February 21st. The career of Dr. Keeley and his remarkable propaganda is one of the peculiar medical anomalies of the closing years of the century. Dr. Keeley in treating the mania for alcohol as a disease introduced nothing novel to medicine. On the contrary, he applied to the treatment of these cases a well-recognized method. Unfortunately, he threw about it a cloak of secrecy and tainted it with humbug and quackery, and brought it into prominence as a great financial scheme. Although at first it was attended with remarkable success, the financial feature proved its undoing. It must be admitted, however, that the Keeley cure has probably served a valuable purpose in disseminating among victims of alcohol a wide recognition of the fact that they are victims of a disease. It has also indirectly promoted temperance by warning people of the danger of contracting an uncontrollable mania. As the vogue of the cure has gradually passed away, it has been replaced by methods of treatment having a reputable medical standing and supplied by regular practitioners.—*Medical News.*

---

THE reports of the suppression of the plague at Honolulu, as stated last week, have been confirmed by despatches dated February 22d. Unfortunately, however, the disease has been introduced in the island Maui. This is one of the Sandwich Islands on which the immense sugar plantations are situated. The seven cases of plague discovered here were in the town Kahului, which is the port of entry of the Island. They all proved fatal, the victims being Chinese or Japanese. The same radical measures were adopted that had formerly been employed at Honolulu. A detention-camp was established and a careful inspection of all persons who had been in that section of the port known as Chinatown was instituted. Chinatown was then destroyed by fire. Investigation by the Board of Health showed that the disease was introduced to Maui through New York delicacies imported from the Orient. On the day that these goods were opened the disease appeared. An effort is now being made to trace the remainder of this consignment. Three of the crew who handled the goods on the steamer which brought them were among the victims. One case has appeared at Hilo, which is the only port at which our transports from the Philippines have called since the outbreak at Honolulu.—*Medical News.*

---

THE London *Lancet*, commenting on the physical condition of the Boers due to their pastoral life and outdoor occupations, says: In so far as they are of Dutch descent, the Boers should be small. The French blood which is mingled with that of the Dutch would not tend very considerably to increase the height of the Boer. The The French, although taller than the Dutch, are not so tall as the English. Yet the Boers of to-day are taller, stronger, and possess a more powerful physique than the English. It is quite a common occurrence to meet a Boer six feet six inches in height. Indeed, it has been said that the average height of the Boer is six feet two inches. All travelers bear witness to their magnificent physique, especially those who have been among the real Boers, that is to say, those who live in the rural districts well away from the railway lines. But apart from the flesh and bone, the big, strong frames, and the hard muscles developed by the healthy, constant outdoor exercise, the Boers have practical freedom from the diseases due to alcoholism and vice. They are not total abstainers, but they are remarkably sober, and drunkenness is rare among them. On the other hand, how often are British soldiers punished for drunkenness and invalided through venereal disease. Not only is almost every Boer physically fit to take the field and fight for his country, but he is a stronger, healthier, and bigger man than even those who have been selected by medical examination as fit to serve in the British army. From this the *Lancet* deduces the lesson that the physical salvation of the human race is to be obtained only by securing at least a reasonable proportion of the fresh air, sunshine and out-of-door life that is enjoyed by the Boer.—*Medical News.*

THE statement recently made by Professor Sumner to a class of 200 senior students in Yale College that 90 per cent. of the marriages of the present time turn out unhappy is an illustration of what nonsense brainy men will sometimes utter. If the question was asked how many couples would probably separate during the first two years of wedded life if neither law nor public opinion discountenanced it, we should say possibly 90 per cent. After the first few weeks of honeymoon come the clash of ideas, the sharp corners of individual character, the smoothing down of which often arouses feelings anything but saintly. This getting thoroughly acquainted with each other often takes one or two years, and during that time there may be an occasional tiff more or less bitter when to each a separation would seem most desirable; but sooner or later there is a better understanding, a closer union, and the erection of a family altar sacred to both, the blending of family ties into a unit in mutual respect and affection. The only separation looked forward to, and that with a shiver of fear, is death. Each has learned that absolute perfection does not exist, and in the closer relations of married life have found in its harmony a rest and a haven from the trouble and the bitterness which so often form a part of our intercourse with the outside world. In the more than fifty years of mingling with the family circle, as physician and friend, with all grades of domestic life, we can safely say that 90 per cent. would testify that there had been more of happiness than unhappiness in the domestic circle, and that married life, in their case, notwithstanding occasional drawbacks, had been to them a blessing for which they were thankful. In doing away with the evils of married life we need less law and more of that domestic training in the home circle based upon love and justice. —*The Medical Times.*

---

RELIGIOUS journals have long been the favorite mediums in which to advertise the innumerable quack remedies and abortifacients which find such an enormous sale among the American people. To many persons an announcement, no matter how preposterous, read in a religious journal, or signed by a clergyman, is at once accepted as a truth not to be disputed. Hence the value of the columns of religious journals to the proprietors of patent medicines and for the personal advertisements of the hordes of charlatans who rival each other in the ingenuity and boldness of their claims to be able to cure all diseases, and who, as these columns are almost always at their disposal, are thus enabled to keep their names constantly before a large and particularly gullible portion of the public. We are informed that *The Christian Herald* has decided that henceforth no advertisements of a medical nature will be admitted to its pages. Whatever motives may have prompted the proprietors of *The Christian Herald* to adopt this policy were certainly good ones, and from a business point of view alone we feel sure that they will have no reason to regret it. We believe that what they may lose in advertising contracts will be much more than gained in subscriptions, and that by purging their pages of what has hitherto befouled them, they will receive the financial support and the good-will of large numbers of sensible and right-minded people. We read with pleasure an editorial on this subject in the *Medical Record* and we heartily agree that "The resolution of *The Christian Herald* to bar altogether medical advertisements from its columns reflects the greatest credit on those in control of that journal, and its example should be promptly followed by other papers whose reading-matter is of a denominational nature." We fear that we cannot hope to see this example followed by the lay press, but we have always believed that a newspaper which announced that it would admit no filthy advertisements, no advertisements which a decent man would not be willing to read aloud to his wife and daughters, would lose nothing by so doing and would probably be the gainer by it.—*St. Paul Medical Journal.*

IN Maryland the Christian Scientists appeared before the Committee on Hygiene of the Legislature in opposition to the proposed bill regulating the practice of medicine. From a report of the proceedings we make a few excerpts concerning which comment is unnecessary :

Dr. Samuel T. Earle asked Mr. Hammond how he discriminated between infectious diseases and those that were not infectious. "The power of God," said Mr. Hammond, "is the same always. We do not believe in infectious diseases, and a person, if a Christian Scientist, could not contract such diseases." Dr. Earle asked whether the method of treatment would be altered for an infectious disease, and Mr. Hammond replied that it would not. Dr. Edward Brush said it was unnecessary to ask the gentleman any further questions, as he had practically admitted that he could bring a patient with the smallpox into a room with other persons, or send a child with scarlet fever to school.

Dr. Earle asked Mrs. Linscott how she could distinguish diphtheria from tonsillitis. Mrs. Linscott laughed heartily and said: "All diseases are the same to us, and we make no microscopic investigations; but I could easily distinguish." Dr. Earle insisted upon knowing by what means, and Mrs. Linscott remaining silent, Mr. Hammond answered in sonorous tone, "Through the power of Almighty God." Mrs. Linscott stated that in a community of 500 people, all of whom were Christian Scientists, such a thing as diphtheria would be impossible, and there would be no necessity for any health department, as the community would be exempt from disease.

Dr. Fulton said the course of medicine of the Scientists was two weeks, according to their own books. He read of a little child who had a terrific toothache and was so completely cured in one night by a Christian Science teacher that not only did the pain leave her, but the cavity of the tooth became filled up, and this was without any physical treatment. The Christian Scientists all exclaimed that this was perfectly true.

Another incident read by Dr. Fulton was to the effect that a lady who had been horribly burned was sufficiently improved to go out the day after the accident. Several of the delegation said they knew this lady.

Another article read referred to a schoolboy who could not do his fractions until aided by Christian Science, when they at once became easy.

Mrs. Linscott remarked to Dr. Fulton: "No wonder the power of God surprises you!"

Dr. Brush read a letter from a member of the Health Department in Buffalo, stating that the sect of Christian Scientists should be suppressed, as they were a menace to the public health.—*Philadelphia Medical Journal.*

